# Serum-Based Lipid Panels for Diagnosis of Idiopathic Parkinson’s Disease

**DOI:** 10.3390/metabo13090990

**Published:** 2023-09-02

**Authors:** Lina A. Dahabiyeh, Refat M. Nimer, Maha Rashed, Jeremiah D. Wells, Oliver Fiehn

**Affiliations:** 1 Department of Pharmaceutical Sciences, School of Pharmacy, The University of Jordan, Amman 11942, Jordan; 2 West Coast Metabolomics Center, University of California, Davis, CA 95616, USA; 3 Department of Medical Laboratory Sciences, Jordan University of Science and Technology, Irbid 22110, Jordan

**Keywords:** Parkinson’s disease, biomarker, lipidomics, oxidative stress, ceramide, PS 40:0, LPC, HETE

## Abstract

Parkinson’s disease (PD) is a highly prevalent neurodegenerative movement disorder with an unclear etiology and a lack of definite diagnostic tests and effective treatments. About 95% of PD cases are idiopathic, in which none of the well-known genes underlying familial parkinsonism are mutated. We used untargeted liquid chromatography–mass spectrometry (LC-MS/MS) to profile the serum lipidome of 50 patients with different stages of idiopathic PD (early, mid, or advanced) and 45 age-matched controls. When comparing the PD patients to the control subjects, 169 lipids were significantly altered in both a univariate analysis and a multivariate partial least-squares discriminant analysis (PLS-DA). Compared to the controls, the patients with PD had higher levels of unsaturated triacylglycerides (e.g., TG O-56:9 and TG 52:3), saturated lysophosphatidylcholines (LPC 17:0, 16:0, and 15:0), and hydroxyeicosatetraenoic acid (12-HETE), while lower levels of phosphatidylserines (e.g., PS 40:4 and PS 16:0_22:4), sphingomyelins (SM 42:1), and ceramides (e.g., Cer 40:0 and 42:0) were found between the PD patients and the controls. A panel of 10 significantly altered lipids (PS 40:0, Cer 40:0, Cer 42:0, LPC 17:0, LPC 15:0, PC 37:7, PE O-40:8, PC O-42:4, FA 23:0, and SM 42:1) resulted in a strong receiver operating characteristic curve with an AUC = 0.974. This panel may, therefore, be useful for diagnosing PD. In addition, lipid panels may prove useful for distinguishing among the progression stages of PD. Using one-way ANOVA, 155 lipid species were significantly altered among the PD stages. Parkinson’s disease progressed from the early to advanced stages with decreasing levels of PC 31:1, PC 38:4, and LPE 22:5. Conversely, LPC-O 20:0, PC O-42:3, FA 19:0, and FA 22:2 showed an increase in their levels with disease progression. Overall, this study shows an intriguing number of robust changes in specific serum lipids that may become useful for diagnosing PD and its progression, once panels have been validated in larger clinical trials and prospective studies.

## 1. Introduction

Parkinson’s disease (PD) is the second most widely spread neurodegenerative disease and the most frequent movement disorder that substantially impacts patient quality of life [1,2]. The main pathological hallmarks of PD are the accumulation of protein α-synuclein, forming aggregates called Lewy bodies, and the degeneration and death of dopaminergic neurons in the nigrostriatal pathway [3]. Besides motor symptoms (e.g., tremors, rigidity, and bradykinesia), patients with PD experience a variety of non-motor features, such as speech and sleep disorders, pain, and fatigue, that become worse with the progression of the disease [4,5]. In the advanced stage of PD, patients will suffer from cognitive disorders, including depression and dementia [4,5].

The majority of PD cases are idiopathic [1]. The lack of specific diagnostic tests continues to challenge the accurate diagnosis of PD. The clinical symptoms of PD are not PD-specific and overlap with other neuropathological disorders, which complicates differential and timely diagnoses [6,7]. Current diagnoses depend mainly on the medical history, a physical examination, and the response to dopaminergic treatments [1]. Moreover, the current treatments are only symptomatic to improve motor and non-motor functions, and no disease-specific therapeutics are currently available [1]. Therefore, there is an urgent need to identify potential diagnostic biomarkers for PD and provide better insights into the underlying disease mechanism(s). This will enhance the current understanding of the pathogenesis of PD and its stages, and aid in the development of disease-modifying therapeutics. 

Lipids have diverse roles in brain function, such as influencing the flexibility and structural arrangement of synaptic membranes as well as regulating the activity of proteins involved in cellular signaling dynamics [8]. In PD, various lipid species are involved in biological processes linked to the disease, including the aggregation potential of protein α-synuclein [9] and mitochondrial functional processes [10]. Additionally, mutations in lipid-producing enzymes have been linked to PD [11]. Recently, the dysregulation of lipid homeostasis in the serum and the cerebrospinal fluid (CSF) has been reported to contribute to the development and progression of PD [12]. 

Lipidomics identifies and quantifies many lipid species across diverse lipid classes, highlighting the alterations in lipid metabolism and lipid-mediated signaling processes [13]. Lipids are directly susceptible to biochemical changes associated with environmental disturbances, pathological processes, or drug treatments. Lipidomics has been applied to study different neurodegenerative disorders, such as multiple sclerosis and Alzheimer’s disease (AD) [14,15]. In PD, different lipidomic platforms use the CSF, serum, plasma, or brain tissue to identify potential markers for the disease, differentiate between PD and AD patients and patients with dementia, and investigate genetic mutations associated with the disease [12,16,17,18]. One study used NMR-based lipidomics to identify PD-stage-specific lipoprotein–lipid signatures in the plasma [16]. These studies revealed a strong correlation between PD and abnormalities in lipid metabolism and the contribution of various subclasses of fatty acyls (saturated and unsaturated fatty acids, a number of eicosanoids, and acylcarnitine), glycerolipids, glycerophospholipids, sphingolipids, sterols, and lipoproteins in PD pathogenesis. Here, we acquired data on serum lipidomic changes using untargeted LC-MS/MS to examine the differences between patients with PD and healthy controls, as well as lipid changes that can inform about disease progression and PD disease severity.

## 2. Materials and Methods

### 2.1. Subjects and Selection Criteria

Ethical approval was obtained from the institutional review board of the Jordan University of Science and Technology (ref.: 26/143/2021, date: 07.09.2021). All participants (patients and controls) provided informed consent before participating in the study.

Patients with idiopathic PD (*n* = 50) were enrolled from public and private neurological clinics and were diagnosed with idiopathic PD by registered neurologists. Healthy controls (*n* = 45) with no clinical evidence of PD or other movement or neurodegenerative disorders were recruited from the local community. Patients with PD that were on medication and without a history of deep brain stimulation were selected [19]. The exclusion criteria included: (i) patients with chronic conditions such as cancer, heart disease, kidney disease, infectious diseases, or nephritis; (ii) patients with neurological disorders, a neurosurgery, a head trauma, dementia, Alzheimer’s disease, or other mental illnesses; (iii) patients with secondary Parkinson’s disease; and (iv) patients on medication different than dopamine analogs or dopamine receptor agonists, such as statins. The patients were classified into three clinical subgroups based on the Hoehn and Yahr staging criteria [20] after a subjective assessment by the examining physician: an early group (H&Y stages I and II) (*n* = 28), a mid group (H&Y stage III) (*n* = 14), and an advanced group (H&Y stages IV and V) (*n* = 8). Patients with seizures and/or metal brain implants were excluded from this study. The healthy controls and patients with PD, as well as patients in the different PD groups, were matched for age, gender, body mass index (BMI), and ethnic origin (Table 1 and Table 2).

### 2.2. Lipid Extraction from Blood Serum

Volumes of 5 mL of blood were collected in plain vacuum blood collection tubes without additives and allowed to clot at room temperature for 30 min. Serum samples were obtained by centrifugation at 1500× *g* for 10 min and then stored as aliquots for analysis at −80 °C. After thawing, 345 µL of cold LC-MS methanol (Fisher Scientific, Hampton, NH, USA) was added to 35 µL of serum, followed by adding 172.5 µL of HPLC chloroform (Fisher Scientific, USA), shaking for 30 s, adding 88 µL of LC-MS water (Fisher Scientific, USA), and then shaking again for 30 s. Equal volumes of chloroform and water were added (172.5 µL), followed by centrifugation at 10,000 rpm for 5 min. From the lower separated organic phase, 250 µL was placed into an Eppendorf tube. All samples were dried using a vacuum centrifugal evaporator (Eppendorf, Hamburg, Germany) and stored at −80 °C until further lipidomics analysis.

### 2.3. Untargeted Lipid Profiling Using Reversed-Phase Liquid Chromatography Coupled with Quadrupole Time-Of-Flight Mass Spectrometry

Dried samples were resuspended in 110 µL of a lipidomics resuspension solution containing 75 internal standards, which included the Avanti UltimateSPLASH™ ONE internal standards kit in addition to several in-house internal standards. The samples were vortexed, sonicated for 5 min, centrifuged at 16,100× *g* for 2 min, and finally, two volumes, each 33 µL, were transferred into two HPLC vials with 200 µL inserts for a separate LC-MS analysis of each polarity mode. Lipid profiling was performed using an Agilent 6530 and Agilent 6550 QTOF MS for the positive and negative mode, respectively. Injected lipid extracts (1.67 and 5 μL for the positive and negative mode, respectively) were separated using a Waters Acquity Premier BEH C18 (1.7 µm, 2.1 × 50 mm) column with the temperature maintained at 65 °C and a flow rate of 0.8 mL/min. The mobile phase for the positive mode was composed of 60:40 *v/v* acetonitrile (ACN):water with 10 mM ammonium formate and 0.1% formic acid as solvent A and 90:10 *v/v* isopropanol:ACN with 10 mM ammonium formate and 0.1% formic acid as solvent B. For the negative mode, 60:40 *v/v* ACN:water with 10 mM ammonium acetate was used as solvent A and 90:10 *v/v* isopropanol:ACN with 10 mM ammonium acetate was used as solvent B. The following gradient was used: 0 min, 15% (B); 0.75 min, 30% (B); 0.98 min, 48% (B); 4.00 min, 82% (B); 4.13 min, 99% (B); 4.50 min, 99% (B); 4.58 min, 15% (B); and 5.50 min, 15% (B).

Data were acquired in the data-dependent acquisition (DDA) mode (10 spectra/s) with the following MS parameters: ESI capillary voltage, +3.5 kV and −3.5 kV; collision energy, 25 eV and 25 eV; and MS1 scan range, 120–1700 Da and 60–1700 Da for the positive and negative mode, respectively. Masshunter 10.1 was used for the data acquisition.

A pooled quality control (QC) sample was prepared by mixing 33 µL of each resuspended sample to assess the performance of the LC-MS instrument. The QC samples were injected every 10 samples and the coefficient of variability (CV) was calculated for all mass ions to ascertain the system suitability and stability.

### 2.4. Data Processing and Lipid Identification 

The raw LC-MS data were processed, for peak picking and alignment, and annotated using the freely available software MS-DIAL, version 4.92 [21]. Thereafter, the data were curated for blank reduction, duplicate and isotope deletion, and finally, adduct combining.

### 2.5. Statistical Analysis (Multivariate and Univariate Analyses)

For the multivariate analysis, the processed datasets (mass ion (Rt, m/z)) pairs with their normalized peak height were imported to Simca P + 14 (Sartorius Stedim Data Analytics AB, Umea, Sweden), mean-centered, and pareto-scaled. A partial least-squares discriminative analysis (PLS-DA) was performed to examine group separation and clustering. The PLS-DA model was validated using cross-validation (leave-one-out method (1 out of 7)) to evaluate the robustness of the generated models by monitoring the fitness of the model using the R2Y value (which indicates the value of the variability in the data explained by the generated model) and the predictive ability of the model using the Q2 value. High R2Y values (close to one) and Q2 values ≥0.5 reflect robust models. The PLS-DA model was also validated using a permutation test (999 permutations). A variable importance in the projection (VIP) >1 was used to extract the important altered lipids in the PLS-DA model [22,23]. 

For the univariate analysis, heat maps, volcano plots, and a receiver operating characteristic (ROC) curve were generated using MetaboAnalyst, version 5.0 (McGill University, Montreal, QC, Canada) [24,25]. The datasets were normalized to the sample’s total median, pareto-scaled, and then subjected to Student’s independent *t*-test (for a PD and control case comparison) or one-way ANOVA (for a comparison among PD stages) to identify significantly altered lipid species among the compared groups. A *p*-value less than 0.05 was defined as significant. Individual lipid abundance comparisons between the control and PD cases and among the PD stages were performed using GraphPad Prism 8 (version 8, San Diego, CA, USA). 

## 3. Results

### 3.1. Demographic and Clinical Data of Participants

The control and PD cases were matched with no significant differences between the two groups regarding age, body mass index (BMI), or gender (Table 1). Similarly, patients in the different PD stages, early, mid, and advanced, were matched with regards to age, BMI, and gender (Table 2).

### 3.2. Lipid Profiling in Patients with PD Compared to Healthy Controls

Around 700 unique lipids were structurally identified from different lipid species, including saturated and unsaturated phosphatidylcholines (PCs), lysoPCs (LPCs), phosphatidylethanolamines (PEs), phosphatidylinositols (PIs), ceramides (Cers), acyl carnitines (ACs), fatty acyl esters of hydroxy fatty acids (FAHFAs), di- and triglycerides (DGs and TGs), and sphingomyelins (SMs) (Figure S1 in Supplementary Information). 

**Figure 1:**
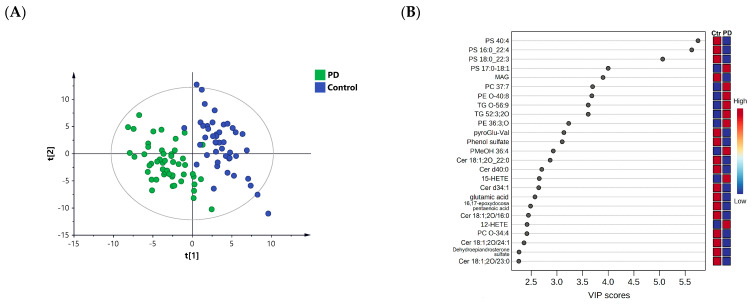
Multivariate analysis of patients with PD (*n* = 50) compared to healthy controls (*n* = 45). (**A**) PLS-DA score plot (R^2^Y = 0.86, Q^2^ = 0.78). (**B**) Top 25 significant lipids and compounds with the highest VIP scores in the PLS-DA model.

To obtain an overview and examine any trends of separation or clustering between the PD and control groups, a PLS-DA score plot was generated (Figure 1A). Evident separation of the two study groups was noticed in the PLS-DA model, reflecting significant changes in the lipidome of the patients with PD compared to the controls. The PLS-DA model yielded satisfactory R2Y (0.86) and Q2 (0.78) values and passed the validity permutation test (Figure S2 in Supplementary Information). 

To extract important altered lipids responsible for the observed class separation in the PLS-DA model, a VIP value > 1 was used. The top lipids accountable for the PLS-DA class separation (VIP >1) included unsaturated phosphatidylserines (PSs) (e.g., PS 40:4, PS 38:4, and PS 40:3), PEs (e.g., PE O-40:8), TGs (e.g., TG O-56:9), Cers (e.g., Cer 40:0 and Cer 34:1), and hydroxyeicosatetraenoic acid products (HETEs; e.g., 12-HETE) (Figure 1B). 

**Figure 2:**
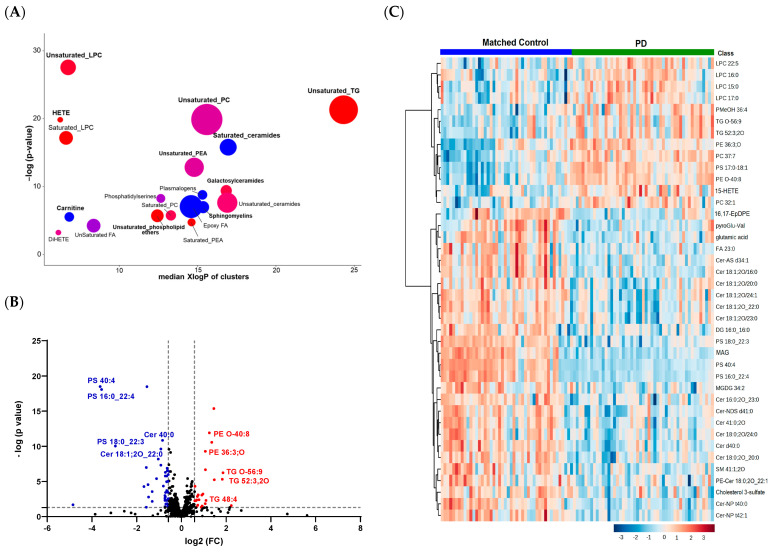
Significantly altered lipid clusters in patients with PD compared to healthy controls. (**A**) ChemRICH enrichment result for significantly impacted lipid clusters. The plot’s *y*-axis shows the most significantly altered clusters on the top. The cluster colors represent the proportion of increased or decreased compounds (red = increased, blue = decreased). (**B**) Volcano plots of up (red)- and down (blue)-regulated lipids using *p*-value and fold-change (FC) cutoffs of <0.05 and 1.5, respectively. (**C**) Heatmap of the top 40 altered lipids. The heatmap was generated based on *t*-test results using autoscale features for the standardization parameter (each compound is autoscaled/z-transformed; thus, each cell displays the feature-wise z-score). The higher values (red) reflect a higher lipid abundance and the lower values (blue) reflect a lower abundance.

A set enrichment analysis using the ChemRICH software [26] revealed that PD impacted several lipid clusters (Figure 2A). Perturbations in unsaturated LPCs, PCs, PEs, and unsaturated Cers were observed in the PD patients. Compared to the controls, the PD cases had higher levels of unsaturated TGs (e.g., TG O-56:9 and TG 52:3), saturated LPCs (LPC 170:0, 16:0, and 15:0), HETEs, and phospholipid ethers, and lower levels of saturated Cers, acyl carnitine, and SMs.

When applying cut-offs of *p* < 0.05, the volcano plot visualization revealed that 65 lipids were dysregulated in the PD patients compared to the controls at fold changes > 1.5 (Figure 2B). Here, we opted for selecting lipids based on the effect sizes (fold changes) in addition to raw *p*-values instead of using Benjamini–Hochberg FDR corrections, because fold changes will be critical when attempting to create robust clinical assays for PD diagnoses. The levels of TG O-56:9, TG 52:3, and PE O-40:8 were significantly increased, while the levels of several PSs (e.g., PS 40:4 and PS 16:0_22:4) and Cer 40:0 were significantly decreased in PD. A visualization of the top perturbed lipid species between the two groups is presented in the heatmap in Figure 2C.

**Figure 3:**
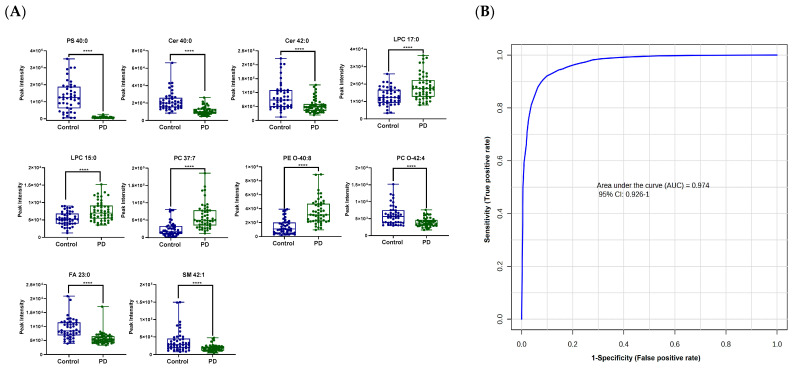
(**A**) Ten significantly altered lipids between control and PD patients (**B**) Receiver operating characteristic (ROC) curve generated from 10 significantly altered lipids between control and PD patients. Significance between the two groups is expressed as ****, which indicates a *p*-value ≤ 0.0001 (Student’s independent *t*-test).

When we combined the results from both the univariate and multivariate analyses, 169 lipids were found to be both significant in the univariate analysis (*p*-value < 0.05) and important for class separation in the multivariate analysis (VIP > 1) (Table S1). These included mainly Cers, LPCs, PCs, PEs, PSs, SMs, and TGs. Ten of the significantly altered lipids, namely PS 40:0, Cer 40:0, Cer 42:0, LPC 17:0, LPC 15:0, PC 37:7, PE O-40:8, PC O-42:4, FA 23:0, and SM 42:1, resulted in an excellent ROC curve with an area-under-the-curve (AUC) value of 0.974 (Figure 3). These ten lipids were manually selected because they represent different biochemical classes and, hence, reflect the dysregulation of different pathways in a more robust way than relying on fewer compounds or fewer pathways. In future validation studies, this list may be modified and expanded to ensure overall robustness across clinical sites. Nevertheless, this initial lipid panel highlights the taunting possibility that a specific assay (using LC–triple quadrupole mass spectrometry, or even specific antibody-based ELISA or dip-stick assays) could be worked out to discriminate between controls and PD cases, and as diagnostic PD biomarkers.

### 3.3. Lipid Profiling of the Three Different Stages of PD: Early, Mid, and Advanced

**Figure 4:**
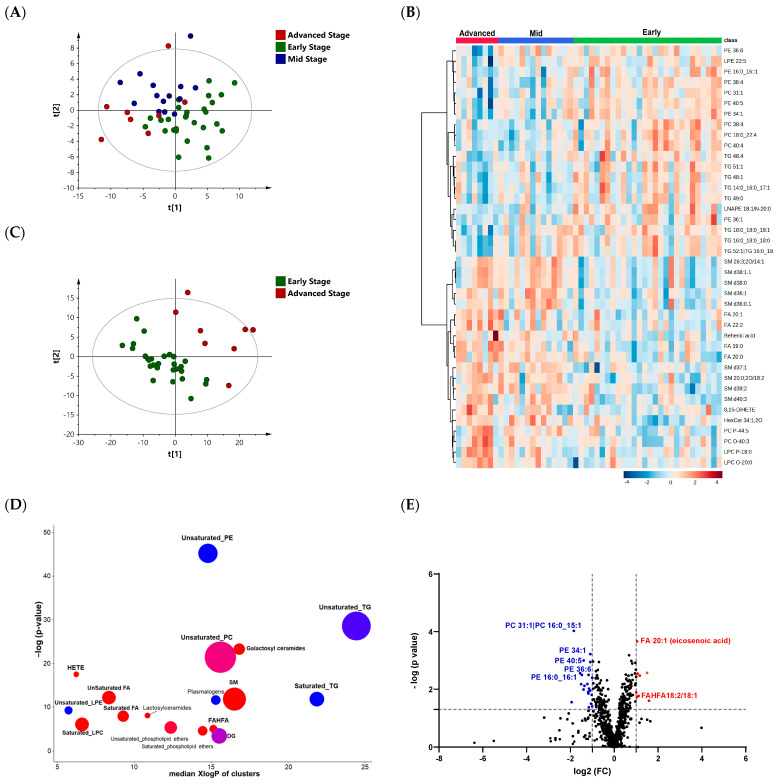
Lipid profiling of different PD stages: early, mid, and advanced. (**A**) PLS-DA score plot (R^2^Y = 0.622, Q^2^ = 0.24) of different PD stages: early (*n* = 28), mid (*n* = 14), and advanced (*n* = 8). (**B**) Heatmap of the top altered lipids. The higher values (red) reflect a higher metabolite abundance, and the lower values (blue) reflect a lower abundance. (**C**) PLS-DA score plot (R^2^Y = 0.784, Q^2^= 0.32) for class separation between early and advanced stages of PD. (**D**) ChemRICH enrichment result for significantly impacted lipid clusters in advanced-stage PD compared with early-stage PD. The plot’s *y*-axis shows the most significantly altered clusters on the top. The cluster colors represent the proportion of increased or decreased compounds (red = increased, blue = decreased). (**E**) Volcano plots of up (red)- and down (blue)-regulated lipids in advanced PD compared with early-stage PD using *p*-value and fold-change (FC) cutoffs of <0.05 and 2, respectively.

The PLS-DA score plot revealed a partial overlap among the three PD stages, where complete separation could not be achieved (Figure 4A). Using one-way ANOVA, 155 lipids were significantly altered among the PD stages (Table S2). The differential lipids were mainly fatty acids, PCs, PEs, LPCs, and SMs. A visualization of the top altered lipids among the three groups is shown in the heat map in Figure 3B.

Among the three PD stages, the lipidomes of the advanced- and early-stage cases were associated with the most significant changes. The PLS-DA score plot revealed complete separation between the two groups (Figure 4C). A ChemRich analysis displayed increased levels of saturated LPCs, HETEs, SMs, and FAHFAs and decreased levels of TGs (saturated and unsaturated) and unsaturated PEs in advanced-stage PD (Figure 4D). The volcano plot with a fold-change cutoff of 2 showed a significant increase and decrease in the levels of 8 and 21 lipids, respectively, in advanced cases compared to early-stage PD (Figure 4E). The levels of eicosenoic acid (FA 20:1) and FAHFA 18:2/18:1 were higher, while the levels of PC 31:1 and several unsaturated PEs (e.g., PE 34:1, PE 40:5) were significantly lower in the advanced stage compared to the early stage (Figure 4E).

**Figure 5:**
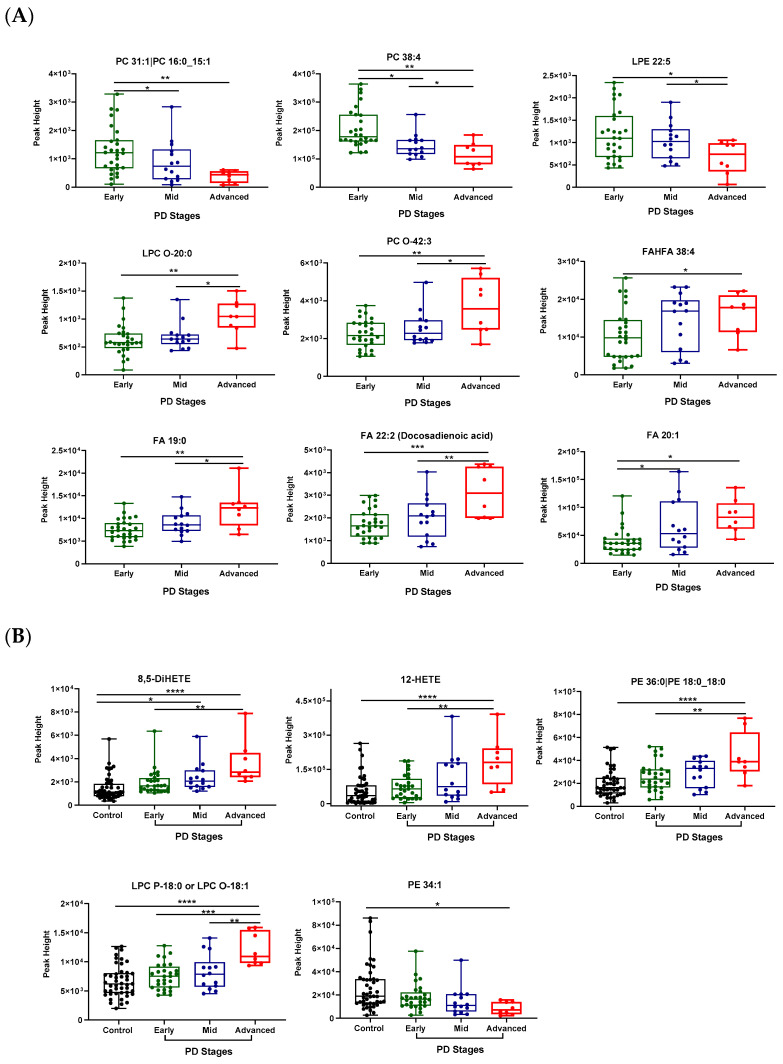
Lipids significantly altered during the progression of PD (**A**) and between controls and PD patients (**B**). One-way ANOVA using Turkey’s post hoc test was used to indicate significance. * *p*-value ≤ 0.05, ** *p*-value ≤ 0.01, *** *p*-value ≤ 0.001, **** *p*-value ≤ 0.0001.

A few lipids such as PC 31:1, PC 38:4, and LPE 22:5 demonstrated a significant decrease in their levels with the progression of PD from the early to advanced stages (Figure 5A). On the other hand, LPC-O 20:0, PC O-42:3, FA 19:0, and FA 22:2 displayed an increase in their levels with disease progression (Figure 5A). Of note, 8,5-DiHETE, 12-HETE, PE 36:0, and LPC O-18:1 were significantly changed between the patients with PD and the controls and among the different stages of PD (Figure 5B).

## 4. Discussion

Parkinson’s disease is the second most common neurodegenerative disorder, and is characterized by the accumulation of α-synuclein, the formation of Lewy bodies in the brainstem, neuroinflammation, oxidative stress, and mitochondrial dysfunction [27]. Around 90–95% of PD cases are defined as idiopathic and have an unknown etiology. Lipids are involved in many aspects of PD pathology, including oxidative stress and inflammation, and lipid dysregulation or disruptions in lipid homeostasis could contribute to the disease pathogenesis. In the presented work, an LC-MS lipidomics approach was applied to analyze serum samples from patients with different stages of PD (early, mid, or advanced) and age-matched controls to identify significantly altered lipids between the PD and control cases and among the different stages of the disease. These lipid signatures might act as potential biomarkers to aid in disease diagnosis and monitoring, provide new insights into the pathophysiology of PD, and identify new therapeutic targets. 

Here, we report significant decreases in the circulating levels of glycerolipids, specifically for both saturated and unsaturated TGs, in advanced-stage PD patients compared to early ones. The mechanisms by which TGs are involved in PD development and progression are unclear, but could be related to their role in neural communication, signal transduction, and cell membrane and mitochondrial functions [28]. Lower serum TG levels were associated with more severe motor performance effects in patients with PD [29]. Our findings indicate that lower TG levels might play a role in accelerating PD progression among early-stage PD individuals, and this could be a potential biomarker for monitoring the progression of the disease. Nevertheless, controversial results regarding the association between serum TG levels and PD have been reported [30,31], warranting further investigation. We also found a significant decrease in the monoacylglyceride (MAG) levels in the patients with PD compared to the controls. Although limited studies are available on the level of MAG in PD patients, studies using PD models suggest that inhibiting MAG lipase, and consequently, increasing the levels of MAG, might have a protective effect against PD [31]. 

Several glycerophospholipid subgroups were altered in PD compared to the controls and during the progression of the disease. Such phospholipids are classified by their polar head groups into PE, PI, PC, PS, and phosphatidylglycerol (PG) lipids. The hydrolysis of one acyl derivative gives rise to the bioactive signaling lipid species of lysophospholipids (LPs) [31]. Interestingly, we found several PSs to be significantly altered in the patients with PD compared to the controls. PSs are involved in neuronal survival and neurotransmitter release [32]. In addition, PSs play a regulatory role in apoptosis and the development of α-synuclein-related pathologies [31]. Our findings support previous studies that report decreased levels of PSs, particularly PS 40:4, in PD patients and in a neuronal cell model of PD [33,34]. 

We found phosphatidylcholines (PCs) to be mostly dysregulated in PD patients when these lipids had long fatty acyl carbon chains with multiple double bonds, e.g., PC 18:2_20:4, PC 38:5, PC 37:7, and PC 36:6, but also peroxisomal-derived ether-linked PC lipid species (PC-O). PCs with longer fatty acyl side chains were found to be increased in a cell model of PD. This increase might be linked to the elevated level of one of the enzymes involved in PC synthesis, phosphocholine cytidylyltransferase, in the substantia nigra of PD patients [34]. 

Specific PCs (PC 31:1, PC 38:4) showed, for the first time, a pattern of decrease in their levels with the progression of PD. PCs are the most abundant glycerophospholipids in cellular and mitochondrial membranes [31]. They provide structural support and play a key role in neuronal processes by impacting the differentiation of neurons and their signaling pathways. They also participate in anti-inflammatory actions [31]. The decrease in the levels of PCs with the progression of PD noticed herein might contribute to the neuroinflammation exacerbation associated with disease advancement. Decreased levels of overall PCs have been described in the substantia nigra of PD patients and in PD animal models [35,36]. 

Our findings revealed an increase in both saturated and unsaturated LPC species in the PD patients compared to the controls and among early- and advanced-stage PD cases. LPCs, the most abundant lysophospholipids in the blood generated by the enzymatic breakdown of PCs, are involved in PD-related pathological processes such as neuroinflammation and oxidative stress [37,38]. Changes in the LPC levels indicate disturbances in the mitochondrial function and defects in their permeability [39]. In a PD cell model, higher LPC levels were associated with cytotoxic changes, a decrease in the mitochondrial potential, and an increase in reactive oxygen species (ROS) formation [40]. The high levels of LPCs, particularly in an advanced stage, might contribute to the neuroinflammation and oxidative stress that are characteristic of PD. Increased levels of LPC 16:0 and 18:1 have been reported in a PD cell model [41], and higher plasma levels of LPC 18:2 have been found in PD patients compared to controls [42]. Similarly, the levels of the previous LPC species were higher in the current work. Besides, LPC O-18:1 was found to be significantly increased in PD patients compared to controls, and its level continued to increase as the disease progressed, suggesting that it can also be used to monitor the progression of PD.

PE phospholipids account for around 45% of the phospholipids in the brain. PEs have a structural role in biological membranes, and they are regulators of cell division [31]. Evidence points towards decreased levels of PEs in PD patients, and lower total PE levels have been observed in the substantia nigra of PD patients [31]. Moreover, in yeast and worm models of PD, a PE deficiency disrupted α-synuclein homeostasis and induced its aggregation [43]. Herein, lower levels of unsaturated PEs were detected in advanced PD compared to early PD. The decrease in the levels of PEs with the progression of the disease could accelerate α-synuclein aggregation and, thus, neurodegeneration. Such a decrease in the PE levels could be due to the increased formation of LPCs from PEs. Nevertheless, the biological implications of the decreased PE levels need to be examined further. Interestingly, two PE species (PE 36:0 and PE 34:1) showed increased and decreased levels in the PD patients compared to the controls and with the advancement of the disease. 

Besides glycerophospholipids, perturbations in the levels of sphingolipids were detected in PD. The levels of several ceramide lipid species were significantly decreased in the PD patients compared to the controls. Cers are bioactive sphingolipids involved in apoptotic pathways and mitochondrial function. Increased and decreased plasma levels of ceramides have been reported in patients with PD [31]. Decreased Cer levels were also reported in the postmortem brain tissue of patients with PD and have been suggested to be potentially pathogenic [44].

Oxidative stress is one of the hallmarks of PD. Higher levels of the eicosanoids 12-HETE and 8,5-DiHETE were noticed in the PD patients compared to the controls and among PD stages, which is consistent with a previous study that reported increased levels of HETEs in the plasma of PD patients [45]. 

PD is a multifactorial heterogeneous disorder that involves several pathophysiological mechanisms and pathways. A diverse and novel panel of lipid species that included ten of the significantly altered lipids (PS 40:0, Cer 40:0, Cer 42:0, LPC 17:0, LPC 15:0, PC 37:7, PE O-40:8, PC O-42:4, FA 23:0, and SM 42:1) possessed a sufficient specificity and sensitivity and yielded a ROC curve with a high classification accuracy. Generally, for any diagnostic technique to be meaningful and acceptable, the AUC must be greater than 0.8 and the lower 95% CI value of the AUC must be >0.5 [46]. The ROC curve generated had an AUC of 0.97 and a lower CI limit of 0.93. This set of promising biomarkers has the potential to function as a diagnostic test for idiopathic PD. Still, it is crucial to validate this finding in a larger cohort, specifically in prospective studies to rule out the potential impact of PD medication itself on the serum lipid levels.

## 5. Conclusions

The etiology and the pathogenesis of PD are still unknown. Our findings provide new insights into the extent of lipid dysfunction in PD that could enhance the development of biomarkers that can assist in the diagnosis of PD and the monitoring of its progression. Notably, the mechanistic links between lipid dysregulation and PD are not understood. One limitation of the study is that, here, we analyzed patient serum levels, which allows for only a modest translation into lipid changes in the brain. Mechanistic studies are best performed in cell, organoid, or animal models. Therefore, future studies that investigate the pathogenesis of PD using cell or animal models remain urgent.

Several serum lipid classes were impacted in PD and during the progression of the disease, including PCs, LPCs, PEs, PSs, Cers, acyl carnitines, HETEs, and TGs. LPC O-18:1, 12-HETE, and PE 36:0 were significantly increased in the PD patients compared to the controls, and their levels continued to increase with disease advancement, suggesting that they can also be used to monitor the progression of PD. The patients in the current work were on PD medications. Although challenging, future studies must include prospective trials with subjects who are not on PD medications, but are later diagnosed with PD, compared to corresponding control subjects to rule out any effects of PD medication that might affect the lipid profile. 

A major challenge in PD is the overlap of its clinical features with those of other neurodegenerative conditions, making misdiagnoses common in idiopathic PD. The panel of ten lipid species offers promising potential biomarkers that might aid in the diagnosis of PD once validated in larger cohorts. Eventually, such lipid panels would be combined into absolute quantification targeted methods such as LC–triple quadrupole MS to evaluate their use as diagnostic tools in clinical settings.

## Figures and Tables

**Table 1:** Demographic data for recruited controls and patients with PD.

Demographic and Clinical Characteristics	PD (*n* = 50)		Control (*n* = 45)		
	Mean	SD	Mean	SD	*p*-Value ^b^
Age (years)	64.2	13.3	59.4	10.4	0.06
Gender (F/M) ^a^	19/31	NA	23/22	NA	0.20
BMI (kg/m^2^)	28.3	5.2	29.9	4.4	0.10
Systolic blood pressure (mmHg)	131.6	16.4	NA	NA	
Diastolic blood pressure (mmHg)	84.2	6.8	NA	NA	
Duration of PD (years)	9.8	7.2	NA	NA	
Total cholesterol (mmol/L)	5.18	0.53	NA	NA	
HDL cholesterol (mmol/L)	1.28	0.25	NA	NA	
LDL cholesterol (mmol/L)	3.45	0.90	NA	NA	

PD: Parkinson’s disease; F/M: female and male; BMI: body mass index. ^a^ Presented as the number of subjects in each group. Values are presented as mean ± SD. ^b^ *p*-value using Student’s *t*-test for age and BMI, and a chi-squared test for gender.

**Table 2:** Demographic data for recruited patients with PD at early, mid, and advanced stages.

Demographic and Clinical Characteristics	PD—Early (*n* = 28)	PD—Mid (*n* = 14)	PD—Advanced (*n* = 8)	*p*-Value ^b^
Age (years)	63.9 ± 12.1	68.7 ± 13.3	57.5 ± 15.8	0.07
Gender (F/M) ^a^	10/18	6/8	3/5	0.12
BMI (kg/m^2^)	28.2 ± 6.4	28.5 ± 2.8	28.3 ± 3.4	0.19

PD: Parkinson’s disease; F/M: female and male; BMI: body mass index. ^a^ Presented as the number of subjects in each group. Values are presented as mean ± SD. ^b^ *p*-value using a one-way ANOVA test for age and BMI, and a chi-squared test for gender.

## Data Availability

The data presented in this study are available from the corresponding author upon request. The data are not publicly available due to privacy reasons.

## Supplementary Materials

The following supporting information can be downloaded at: https://www.mdpi.com/article/10.3390/metabo13090990/s1. Figure S1: Main lipid classes identified in the study. Figure S2: Permutation tests for the validation of the PLS-DA model generated from the comparison of the serum lipidome of PD patients to those of healthy control cases. A permutation test was performed with 999 random permutations. R2Y (blue squares) and Q2 (green circles) values from the permuted analysis (bottom left) are significantly lower than the corresponding original values (top right). Table S1: Summary of significantly altered lipids (in multivariate and univariate analyses) in Parkinson’s disease patients compared to healthy controls. Table S2: Summary for significantly altered lipids among different Parkinson’s disease stages (early, mid, and advanced), as calculated by one-way ANOVA, with their *p*-values.

## Abbreviations

AC: acyl carnitine; ACN: acetonitrile; AD: Alzheimer’s disease; ANOVA: analysis of variance; AUC: area under the curve; BMI: body mass index; Cer: ceramide; CI: confidence interval; CSF: cerebrospinal fluid; CV: coefficient of variability; DDA: data-dependent acquisition; DG: diglyceride; DiHETE: dihydroxyeicosatetraenoic acid; ELISA: enzyme-linked immunosorbent assay; ESI: electrospray ionization; FA: fatty acyl ester; FAHFA: fatty acyl ester of hydroxy fatty acids; FC: fold change; FDR: false discovery rate; HETE: hydroxyeicosatetraenoic acid; LC-MS: liquid chromatography–mass spectrometry; LPC: lysophosphatidylcholine; MAG: monoacylglyceride; MS: mass spectrometry; NMR: nuclear magnetic resonance; PC: phosphatidylcholine; PC-O: peroxisomal-derived ether-linked PC lipid species; PD: Parkinson’s disease; PE: phosphatidylethanolamine; PI: phosphatidylinositol; PLS-DA: partial least-squares discriminant analysis; PS: phosphatidylserine; QC: quality control; QTOF MS: quadrupole time-of-flight mass spectrometry; ROC: receiver operating characteristic; ROS: reactive oxygen species; SM: sphingomyelin; TG: triglyceride; VIP: variable importance in the projection.

## References

[1] Armstrong M.J., Okun M.S. (2020). Diagnosis and Treatment of Parkinson Disease A Review. JAMA J. Am. Med. Assoc..

[2] Dorsey E.R., Elbaz A., Nichols E., Abd-Allah F., Abdelalim A., Adsuar J.C., Ansha M.G., Brayne C., Choi J.Y.J., Collado-Mateo D. (2018). Global, regional, and national burden of Parkinson’s disease, 1990–2016: A systematic analysis for the Global Burden of Disease Study 2016. Lancet Neurol..

[3] Simon D.K., Tanner C.M., Brundin P. (2020). Parkinson Disease Epidemiology, Pathology, Genetics, and Pathophysiology. Clin. Geriatr. Med..

[4] Jankovic J. (2008). Parkinson’s disease: Clinical features and diagnosis. J. Neurol. Neurosurg. Psychiatry.

[5] Varadi C. (2020). Clinical Features of Parkinson’s Disease: The Evolution of Critical Symptoms. Biology.

[6] Schrag A., Horsfall L., Walters K., Noyce A., Petersen I. (2015). Prediagnostic presentations of Parkinson's disease in primary care: A case-control study. Lancet Neurol..

[7] Berg D., Postuma R.B., Adler C.H., Bloem B.R., Chan P., Dubois B., Gasser T., Goetz C.G., Halliday G., Joseph L. (2015). MDS research criteria for prodromal Parkinson’s disease. Mov. Disord..

[8] Lauwers E., Goodchild R., Verstreken P. (2016). Membrane Lipids in Presynaptic Function and Disease. Neuron.

[9] Galvagnion C. (2017). The Role of Lipids Interacting with alpha-Synuclein in the Pathogenesis of Parkinson’s Disease. J. Park. Dis..

[10] Hu Q.S., Wang G.H. (2016). Mitochondrial dysfunction in Parkinson’s disease. Transl. Neurodegener..

[11] Fanning S., Selkoe D., Dettmer U. (2021). Vesicle trafficking and lipid metabolism in synucleinopathy. Acta Neuropathol..

[12] Galper J., Dean N.J., Pickford R., Lewis S.J.G., Halliday G.M., Kim W.S., Dzamko N. (2022). Lipid pathway dysfunction is prevalent in patients with Parkinson’s disease. Brain.

[13] Spener F., Lagarde M., Geloen A., Record M. (2003). What is lipidomics?. Eur. J. Lipid Sci. Technol..

[14] Pieragostino D., Cicalini I., Lanuti P., Ercolino E., di Ioia M., Zucchelli M., Zappacosta R., Miscia S., Marchisio M., Sacchetta P. (2018). Enhanced release of acid sphingomyelinase-enriched exosomes generates a lipidomics signature in CSF of Multiple Sclerosis patients. Sci. Rep..

[15] Liu Y., Thalamuthu A., Mather K.A., Crawford J., Ulanova M., Wong M.W.K., Pickford R., Sachdev P.S., Braidy N. (2021). Plasma lipidome is dysregulated in Alzheimer’s disease and is associated with disease risk genes. Transl. Psychiatry.

[16] Pizarro C., Esteban-Diez I., Espinosa M., Rodriguez-Royo F., Gonzalez-Saiz J.M. (2019). An NMR-based lipidomic approach to identify Parkinson’s disease-stage specific lipoprotein-lipid signatures in plasma. Analyst.

[17] Chiurchiu V., Tiberi M., Matteocci A., Fazio F., Siffeti H., Saracini S., Mercuri N.B., Sancesario G. (2022). Lipidomics of Bioactive Lipids in Alzheimer’s and Parkinson’s Diseases: Where Are We?. Int. J. Mol. Sci..

[18] Fernandez-Irigoyen J., Cartas-Cejudo P., Iruarrizaga-Lejarreta M., Santamaria E. (2021). Alteration in the Cerebrospinal Fluid Lipidome in Parkinson’s Disease: A Post-Mortem Pilot Study. Biomedicines.

[19] Hacker M.L., DeLong M.R., Turchan M., Heusinkveld L.E., Ostrem J.L., Molinari A.L., Currie A.D., Konrad P.E., Davis T.L., Phibbs F.T. (2018). Effects of deep brain stimulation on rest tremor progression in early stage Parkinson disease. Neurology.

[20] Hoehn M.M., Yahr M.D. (1967). Parkinsonism: Onset, progression, and mortality. Neurology.

[21] Tsugawa H., Cajka T., Kind T., Ma Y., Higgins B., Ikeda K., Kanazawa M., VanderGheynst J., Fiehn O., Arita M. (2015). MS-DIAL: Data-independent MS/MS deconvolution for comprehensive metabolome analysis. Nat. Methods.

[22] Chong I.G., Jun C.H. (2005). Performance of some variable selection methods when multicollinearity is present. Chemom. Intell. Lab. Syst..

[23] Banerjee P., Ghosh S., Dutta M., Subramani E., Khalpada J., RoyChoudhury S., Chakravarty B., Chaudhury K. (2013). Identification of Key Contributory Factors Responsible for Vascular Dysfunction in Idiopathic Recurrent Spontaneous Miscarriage. PLoS ONE.

[24] Pang Z., Chong J., Zhou G., de Lima Morais D.A., Chang L., Barrette M., Gauthier C., Jacques P.-É., Li S., Xia J. (2021). MetaboAnalyst 5.0: Narrowing the gap between raw spectra and functional insights. Nucleic Acids Res..

[25] Xia J., Wishart D.S. (2011). Web-based inference of biological patterns, functions and pathways from metabolomic data using MetaboAnalyst. Nat. Protoc..

[26] Barupal D.K., Fiehn O. (2017). Chemical Similarity Enrichment Analysis (ChemRICH) as alternative to biochemical pathway mapping for metabolomic datasets. Sci. Rep..

[27] Alecu I., Bennett S.A.L. (2019). Dysregulated Lipid Metabolism and Its Role in alpha-Synucleinopathy in Parkinson’s Disease. Front. Neurosci..

[28] Gomez-Soler M., Cordobilla B., Morato X., Fernandez-Duenas V., Domingo J.C., Ciruela F. (2018). Triglyceride Form of Docosahexaenoic Acid Mediates Neuroprotection in Experimental Parkinsonism. Front. Neurosci..

[29] Zhang M.M., Chen H.M., Liu G.L., Wang X.M., Wang Z., Feng T., Zhang Y.M. (2022). Lower serum triglyceride levels linked to more severe motor performance in Parkinson’s disease. Neurol. Sci..

[30] Huang X.X., Ng S.Y.E., Chia N.S.Y., Acharyya S., Setiawan F., Lu Z.H., Tan Y.J., Ng E., Wen M.C., Ng A.S.L. (2018). Higher serum triglyceride levels are associated with Parkinson’s disease mild cognitive impairment. Mov. Disord..

[31] Xicoy H., Wieringa B., Martens G.J.M. (2019). The Role of Lipids in Parkinson’s Disease. Cells.

[32] Kim H.Y., Huang B.X., Spector A.A. (2014). Phosphatidylserine in the brain: Metabolism and function. Prog. Lipid Res..

[33] Zhang J.Z., Zhang X., Wang L.J., Yang C.D. (2017). High Performance Liquid Chromatography-Mass Spectrometry (LC-MS) Based Quantitative Lipidomics Study of Ganglioside-NANA-3 Plasma to Establish Its Association with Parkinson’s Disease Patients. Med. Sci. Monit..

[34] Xicoy H., Brouwers J.F., Kalnytska O., Wieringa B., Martens G.J.M. (2020). Lipid Analysis of the 6-Hydroxydopamine-Treated SH-SY5Y Cell Model for Parkinson’s Disease. Mol. Neurobiol..

[35] Seyfried T.N., Choi H., Chevalier A., Hogan D., Akgoc Z., Schneider J.S. (2018). Sex-Related Abnormalities in Substantia Nigra Lipids in Parkinson’s Disease. ASN Neuro.

[36] Farmer K., Smith C.A., Hayley S., Smith J. (2015). Major Alterations of Phosphatidylcholine and Lysophosphotidylcholine Lipids in the Substantia Nigra Using an Early Stage Model of Parkinson’s Disease. Int. J. Mol. Sci..

[37] Hung N.D., Sok D.E., Kim M.R. (2012). Prevention of 1-palmitoyl lysophosphatidylcholine-induced inflammation by polyunsaturated acyl lysophosphatidylcholine. Inflamm. Res..

[38] Law S.H., Chan M.L., Marathe G.K., Parveen F., Chen C.H., Ke L.Y. (2019). An Updated Review of Lysophosphatidylcholine Metabolism in Human Diseases. Int. J. Mol. Sci..

[39] Hollie N.I., Cash J.G., Matlib A., Wortman M., Basford J.E., Abplanalp W., Hui D.Y. (2014). Micromolar changes in lysophosphatidylcholine concentration cause minor effects on mitochondrial permeability but major alterations in function. Biochim. Biophys. Acta Mol. Cell Biol. Lipids.

[40] Lee E.S.Y., Chen H., Charlton C.G., Soliman K.F.A. (2005). The role of phospholipid methylation in 1-methyl-4-phenyl-pyridinium ion (MPP+)-induced neurotoxicity in PC12 cells. Neurotoxicology.

[41] Lobasso S., Tanzarella P., Vergara D., Maffia M., Cocco T., Corcelli A. (2017). Lipid profiling of parkin-mutant human skin fibroblasts. J. Cell. Physiol..

[42] Zhao H.Y., Wang C., Zhao N., Li W.X., Yang Z.F., Liu X.X., Le W.D., Zhang X.Z. (2018). Potential biomarkers of Parkinson’s disease revealed by plasma metabolic profiling. J. Chromatogr. B Anal. Technol. Biomed. Life Sci..

[43] Wang S.X., Zhang S.Y., Liou L.C., Ren Q., Zhang Z.J., Caldwell G.A., Caldwell K.A., Witt S.N. (2014). Phosphatidylethanolamine deficiency disrupts alpha-synuclein homeostasis in yeast and worm models of Parkinson disease. Proc. Natl. Acad. Sci. USA.

[44] Abbott S.K., Li H.Y., Munoz S.S., Knoch B., Batterham M., Murphy K.E., Halliday G.M., Garner B. (2014). Altered ceramide acyl chain length and ceramide synthase gene expression in Parkinson’s disease. Mov. Disord..

[45] Seet R.C.S., Lee C.Y.J., Lim E.C.H., Tan J.J.H., Quek A.M.L., Chong W.L., Looi W.F., Huang S.H., Wang H.S., Chan Y.H. (2010). Oxidative damage in Parkinson disease: Measurement using accurate biomarkers. Free Radic. Biol. Med..

[46] Nahm F.S. (2022). Receiver operating characteristic curve: Overview and practical use for clinicians. Korean J. Anesthesiol..

