# Supplementary material for: Serum-Based Lipid Panels for Diagnosis of Idiopathic Parkinson’s Disease
Source: Metabolites. 2023 Sep 2;13(9):990. doi: 10.3390/metabo13090990 (PMC10537766; doi:10.3390/metabo13090990)
Supplement: Supplementary file 1 [file metabolites-13-00990-s001.zip › Supplementary Figures_Figure S1 and S2.pdf]

List of Supplementary Figures

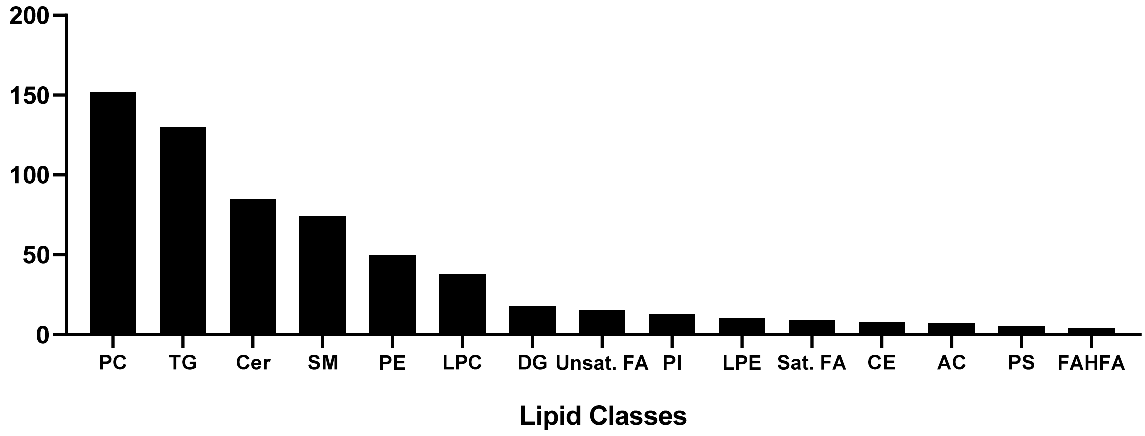

Figure S1. Main Lipid Classes identified in the study

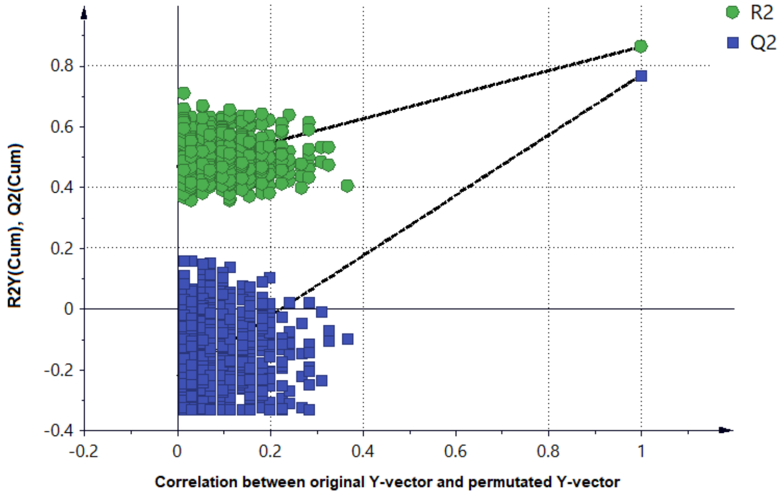

Figure S2. Permutation tests for the validation of the PLS-DA model generated from the comparison of serum lipidome of PD to health control cases. A permutation test was performed with 999 random permutations. R^2Y (blue squares) and Q^2 (green circles) values from the permuted analysis (bottom left) are significantly lower than the corresponding original values (top right).
